# A Comparison of Oxygenation Efficacy between High-Flow Nasal Cannulas and Standard Facemasks during Elective Tracheal Intubation for Patients with Obesity: A Randomized Controlled Trial

**DOI:** 10.3390/jcm11061700

**Published:** 2022-03-18

**Authors:** Yu-Ming Wu, Chun-Cheng Li, Shih-Yu Huang, Yen-Hao Su, Chien-Wun Wang, Jui-Tai Chen, Shih-Chiang Shen, Po-Han Lo, Yun-Ling Yang, Yih-Giun Cherng, Hsiang-Ling Wu, Ying-Hsuan Tai

**Affiliations:** 1 Department of Anesthesiology, Shuang Ho Hospital, Taipei Medical University, New Taipei City 23561, Taiwan; 15538@s.tmu.edu.tw (Y.-M.W.); 15193@s.tmu.edu.tw (C.-C.L.); 10689@s.tmu.edu.tw (S.-Y.H.); 17117@s.tmu.edu.tw (C.-W.W.); 19240@s.tmu.edu.tw (J.-T.C.); 12006@s.tmu.edu.tw (P.-H.L.); 10500@s.tmu.edu.tw (Y.-L.Y.); stainless@s.tmu.edu.tw (Y.-G.C.); 2 Department of Anesthesiology, School of Medicine, College of Medicine, Taipei Medical University, Taipei 11031, Taiwan; 3 Division of General Surgery, Department of Surgery, Shuang Ho Hospital, Taipei Medical University, New Taipei City 23561, Taiwan; su11066@tmu.edu.tw (Y.-H.S.); 17199@s.tmu.edu.tw (S.-C.S.); 4 Department of Surgery, School of Medicine, College of Medicine, Taipei Medical University, Taipei 11031, Taiwan; 5 Department of Anesthesiology, Taipei Veterans General Hospital, Taipei 11217, Taiwan; hlwu9@vghtpe.gov.tw; 6 School of Medicine, National Yang Ming Chiao Tung University, Taipei 11221, Taiwan

**Keywords:** apneic oxygenation, high-flow nasal cannula, hypoxemia, oxygen therapy, rapid sequence intubation

## Abstract

Obese patients are predisposed to rapid oxygen desaturation during tracheal intubation. We aimed to compare the risk of desaturation between high-flow nasal oxygenation (HFNO) and classical facemask oxygenation (FMO) during rapid sequence intubation for elective surgery in obese patients. Adults with a body mass index ≥30 kg·m^−2^ undergoing laparoscopic sleeve gastrectomy at a medical center were randomized into the HFNO group (*n* = 40) and FMO group (*n* = 40). In the HFNO group, patients used a high-flow nasal cannula to receive 30 to 50 L·min^−1^ flow of heated and humidified 100% oxygen. In the FMO group, patients received a fitting facemask with 15 L·min^−1^ flow of 100% oxygen. After 5-min preoxygenation, rapid sequence intubation was performed. The primary outcome was arterial desaturation during intubation, defined as a peripheral capillary oxygen saturation (SpO_2_) <92%. The risk of peri-intubation desaturation was significantly lower in the HFNO group compared to the FMO group; absolute risk reduction: 0.20 (95% confidence interval: 0.05–0.35, *p* = 0.0122); number needed to treat: 5. The lowest SpO_2_ during intubation was significantly increased by HFNO (median 99%, interquartile range: 97–100) compared to FMO (96, 92–100, *p* = 0.0150). HFNO achieved a higher partial pressure of arterial oxygen (PaO_2_) compared to FMO, with medians of 476 mmHg (interquartile range: 390–541) and 397 (351–456, *p* = 0.0010), respectively. There was no difference in patients’ comfort level between groups. Compared with standard FMO, HFNO with apneic oxygenation reduced arterial desaturation during tracheal intubation and enhanced PaO_2_ among patients with obesity.

## 1. Introduction

Obesity is a global epidemic, affecting about 13% of the adult population worldwide [1]. In addition, the global prevalence of obesity has nearly tripled from 1975 to 2016 [1]. Obesity diminishes almost every aspect of health, from the cardiovascular and respiratory systems to the musculoskeletal system [2,3]. Accordingly, the number of obese patients requiring surgical procedures and anesthesia is projected to increase due to the growing prevalence of obesity and the comorbid diseases related to obesity [4,5].

Obese patients pose a considerable challenge for anesthesia care practice due to their poor respiratory reserve and rapid hemoglobin desaturation during apnea [6]. Obesity has various deleterious effects on pulmonary functions, including reduced vital capacity, inspiratory capacity, expiratory reserve volume, functional residual capacity, and lower lung compliance [7]. Moreover, obesity is also the most important risk factor for obstructive sleep apnea [8], which predisposes patients to difficult mask ventilation and rapid oxygen desaturation during tracheal intubation [9]. Studies have shown that the nonhypoxic apnea time of peripheral oxygen saturation (SpO_2_) dropping to 90% after facemask preoxygenation is less than 3 min in obese patients, compared to 6 min in those with a normal body mass index (BMI) [10].

High-flow nasal cannulas provide warmed and humidified airflow and enable oxygen to be comfortably delivered at a rate over 60 L·min^−1^ [11]. In procedural sedation, high-flow nasal cannula is commonly used to protect patients against the occurrence of hypoxemia [12,13]. However, few studies have compared the efficacy of high-flow nasal oxygenation (HFNO) and standard facemask oxygenation (FMO) in preventing desaturation and enhancing arterial oxygen levels during tracheal intubation for obese patients [14,15,16,17,18] Three clinical trials demonstrated that HFNO prolonged nonhypoxic apnea time and increased partial pressure of arterial oxygen (PaO_2_) as a preoxygenation technique for obese patients [14,15,18]. By contrast, another trial claimed that HFNO carried a higher risk of desaturation during intubation and offered a lower end-tidal oxygen concentration after intubation in obese patients [16]. A post hoc analysis of a randomized trial recently reported that obese patients with hypoxemic respiratory failure experienced a similar incidence of peri-intubation hypoxemia (SpO_2_ < 80%) between HFNO and FMO [17]. It remains undetermined whether HFNO is a practicable alternative to classic FMO in reducing peri-intubation desaturation and enhancing arterial oxygenation in obese populations. In general, the current evidence is sparse and controversial.

We conducted a single-center randomized controlled trial to compare the efficacy of preoxygenation between HFNO and FMO for obese patients undergoing general anesthesia. Specifically, we hypothesized that HFNO reduces the risk of desaturation during tracheal intubation and achieves a higher PaO_2_ in obese patients.

## 2. Materials and Methods

### 2.1. Criteria of Patient Selection

This study was reviewed and approved by the Institutional Review Board of Taipei Medical University in Taiwan (TMU-JIRB-N202002076). It was registered in an international directory, www.clinicaltrials.gov (accessed on 27 September 2021) (identifier: NCT04395248). All participants provided oral and written informed consent before randomization. This trial was performed in accordance with the Helsinki Declaration and relevant regulations.

We conducted a two-arm open-label randomized controlled trial to prospectively enroll patients who underwent laparoscopic sleeve gastrectomy at a medical center between May 2020 and August 2021. Inclusion criteria were: age 20 to 65 years, and BMI equal to or higher than 30 kg·m^−2^. Exclusion criteria were: SpO_2_ < 90% in room air, severe cardiopulmonary disease (e.g., left ventricular ejection fraction < 40%, diagnosed coronary artery disease, and aortic dissection), hemodynamic instability, renal insufficiency (estimated glomerular filtration rate <30 mL·min·1.73 m^−2^), pregnancy, and patient refusal.

### 2.2. Randomization Methods

Participants were randomized into the HFNO group and FMO group in a ratio of 1:1. The RAND function of Statistics Analysis System (SAS), version 9.4 (SAS Institute Inc., Cary, NC, USA) was implemented to generate block randomization. After obtaining the informed consent, each participant was given a unique identifier and a group assignment by the principal investigator. The assignment was then enclosed in envelopes and sealed. An attending anesthesiologist (Y.-M.W. or S.-Y.H.) opened the prepared envelopes upon patients’ arrival at the operating room and administered the assigned intervention.

### 2.3. Protocol of Preoxygenation

Each patient was placed in the ramped position and then moved into the reverse Trendelenburg position to achieve a 30-degree incline of the thorax before oxygen therapy. In the HFNO group, preoxygenation was performed using a high-flow nasal cannula (Optiflow™, Fisher & Paykel Healthcare, Auckland, New Zealand), with nasal prongs set at 30 L·min^−1^ flow of heated and humidified 100% oxygen. In the FMO group, patients breathed spontaneously with a size-3 or -4 fitting anesthetic facemask (PAHSCO Inc., Miaoli, Taiwan) connected to a ventilation system (Carestation 620, Datex-Ohmeda Inc., Madison, WI, USA) with 100% oxygen 15 L·min^−1^. The gas flow of HFNO and FMO was adjusted depending on patients’ tolerance.

After preoxygenation for 5 min, rapid sequence intubation was performed in all patients, as follows. General anesthesia was induced with propofol 1.5–2.0 mg·kg^−1^ ideal body weight and fentanyl 2–3 μg·kg^−1^ total body weight. After the abolition of eyelash reflex, rocuronium 0.8–1.0 mg·kg^−1^ ideal body weight was administered and immediately followed by a flush of 20 mL normal saline for a rapid neuromuscular blockade [19]. Meanwhile, upper airway patency was maintained with a two-handed jaw-thrust technique in both groups, and the oxygen flow of nasal prongs was escalated to 50 L·min^−1^ in the HFNO group. At 1 min after rocuronium infusion, direct laryngoscopy intubation was performed by the attending anesthesiologist as the intubator and two senior nurse anesthetists as the assistants. The tracheal intubation was performed using a size-3 or -4 Macintosh blade (Rüsch Inc., Duluth, GA, USA) and a 7.5- or 8.0-mm styleted endotracheal tube (Unomedical, ConvaTec Inc., Deeside, Wales, UK). Accumulating evidence has supported the potential benefits of apneic oxygenation using high-flow nasal cannulas in preventing desaturation for patients with obesity [14,15,18,20,21]. Accordingly, during intubation, the nasal prongs of the HFNO group were left in place in order to achieve apneic oxygenation. In the FMO group, the facemask was removed during intubation. In case of failed direct laryngoscopy despite external laryngeal manipulation, GlideScope^®^ (Verathon Medical, Bothell, WA, USA) was allowed as an intubation rescue technique at the discretion of the anesthesiologist. The correct placement of the endotracheal tube was confirmed by end-tidal capnography, and the nasal prongs were then removed in the HFNO group. Patients were administered 100% oxygen, and a recruitment maneuver was promptly performed with peak airway pressure 40 cm H_2_O for 10 s until the SpO_2_ was restored to the baseline value.

### 2.4. Study Outcomes

The primary outcome was arterial oxygen desaturation, defined as the SpO_2_ < 92% during tracheal intubation. The secondary outcomes were the lowest SpO_2_ during intubation, PaO_2_, partial pressure of arterial carbon dioxide (PaCO_2_), SpO_2_ after preoxygenation, and patient comfort levels. Arterial catheters were placed before the induction of anesthesia. Arterial blood gas was measured twice: first, before the preoxygenation in room air; second, just after the 5-min preoxygenation. The patients and anesthesiologists performing the tracheal intubation could not be blinded. An independent nurse anesthetist (Y.-L.Y.) as the outcome adjudicator read the capnography and SpO_2_ and determined the success of tracheal intubation and the lowest SpO_2_ during intubation. After surgery, the patient’s comfort levels of HFNO and FMO were assessed using a 10-point Likert scale at the post-anesthesia care unit by the same adjudicator.

### 2.5. Sample Size Estimation

According to a recent meta-analysis, at least 35 patients in each group of HFNO and FMO were needed to detect an odds ratio of 0.06 of oxygen desaturation between the two techniques, accepting a type I error of 5% and type II error of 20% with an anticipated desaturation rate of 0.37 in the FMO group [17,22,23]. This study enrolled 40 patients in each group to compensate for possible dropouts due to intolerance of oxygenation interventions.

### 2.6. Statistical Analysis

Shapiro–Wilk and Kolmogorov–Smirnov tests were used to examine the normality of included variables. Normally distributed variables were expressed as mean ± standard deviation. Non-normally distributed data were presented as median with interquartile range, minimum, and maximum. The distributions of baseline patient characteristics and outcome variables were compared between the HFNO group and FMO group using chi-square tests or Fisher’s exact tests for categorical variables and either *t*-tests or Mann–Whitney U tests for continuous variables, as appropriate. For sensitivity analyses, the rates of the minimum SpO_2_ < 90% and 95% were also compared between the two groups. In addition, an analysis excluding patients who needed more than one attempt for successful tracheal intubation was also conducted. Subgroup analyses by BMI and obstructive sleep apnea were performed to examine the preoxygenation efficacy of HFNO versus FMO among these strata. We considered *p* < 0.05 statistically significant for a two-sided test. All the statistical analyses were conducted using SAS software.

## 3. Results

### 3.1. Baseline Patient Characteristics

**Figure 1:**
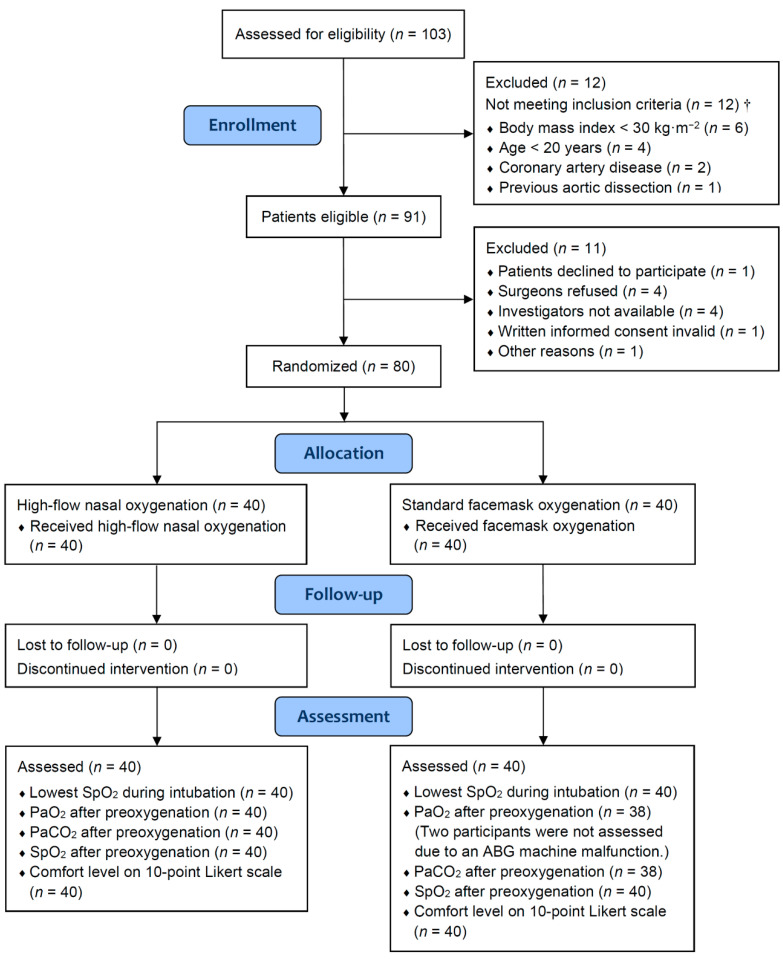
Consolidated Standards of Reporting Trials flow diagram. Abbreviations: ABG, arterial blood gas; PaCO_2_, partial pressure of arterial carbon dioxide; PaO_2_, partial pressure of arterial oxygen; SpO_2_, peripheral capillary oxygen saturation. † Not mutually exclusive because patients can have more than one exclusion criterion.

After meeting the selection criteria, 80 patients were enrolled and randomized into the HFNO group (*n* = 40) and FMO group (*n* = 40) (Figure 1). There was no difference in the baseline patient characteristics between the two groups (Table 1). There was no difference in the SpO_2_, PaO_2_, or PaCO_2_ before preoxygenation between groups, either (Table 2). In addition, the doses of intravenous anesthetics were similar between groups. The first-attempt success rate of intubation and time to successful intubation were also comparable between groups. In all patients, the trachea was intubated successfully by direct laryngoscopy. No sign of regurgitation of gastric content was detected.

### 3.2. Oxygenation Efficacy

The risk of peri-intubation desaturation was significantly lower in the HFNO group (*n* = 2, 5.0%) compared to the FMO group (*n* = 10, 25.0%, *p* = 0.0122); absolute risk reduction: 0.20 [95% confidence interval (CI): 0.05–0.35]; number needed to treat: 5. The lowest SpO_2_ during intubation was significantly increased by HFNO (median 99%, interquartile range: 97–100) compared to FMO (96%, 92–100, *p* = 0.0150); mean difference: 3% (95% CI: 1–5). In addition, HFNO achieved a higher PaO_2_ after preoxygenation compared to FMO: median 476 mm Hg (interquartile range: 390–541) and 397 mm Hg (351–456, *p* = 0.0010); mean difference 73 mm Hg (95% CI: 32–114). There was no difference in the PaCO_2_ or SpO_2_ after preoxygenation, or patient comfort levels between groups (Table 3). The results of the sensitivity analyses are shown in Table 4.

### 3.3. Subgroup Analyses

The reduced desaturation rate and the increased minimum SpO_2_ during tracheal intubation in the HFNO group were significant in the subgroups of BMI ≥ 40 kg·m^−2^ and no obstructive sleep apnea. By contrast, the higher PaO_2_ after preoxygenation in HFNO was significant in patients with a BMI < 40 kg·m^−2^ (Table 5).

## 4. Discussion

Compared to classical FMO, this trial demonstrated that HFNO with apneic oxygenation reduced the risk of oxygen desaturation and enhanced the lowest SpO_2_ during tracheal intubation for obese patients. Additionally, HFNO used as a preoxygenation technique achieved a higher PaO_2_ while offering similar levels of patient comfort compared to FMO. The subgroup analyses further showed that the decreased oxygen desaturation and the increased minimum SpO_2_ during intubation by HFNO were significant exclusively among morbidly obese patients. Noticeably, these clinical benefits were demonstrated in the setting of rapid sequence intubation. Given the higher risks of rapid oxygen desaturation and difficult ventilation in obese population, our results might provide an important clinical implication for preventing hypoxemia and allowing more time to establish an airway.

There are still few studies that compare the efficacy of HFNO and FMO in preoxygenation for anesthetized obese patients [14,15,16,17,18]. The present study showed that HFNO prevented the occurrence of arterial desaturation during tracheal intubation, agreeing with two studies [15,18] but not another [16]. Wong and colleagues reported that HFNO prolonged the nonhypoxic apnea time (SpO_2_ > 95%) by 76 s and increased the lowest SpO_2_ in morbidly obese patients [15]. By contrast, Vourc’h and co-workers revealed that HFNO produced a lower end-tidal oxygen concentration after intubation and carried a higher risk of desaturation below 95% [16]. These discrepancies might be explained by the differences in the oxygenation protocol and measurement of desaturation. In the study of Vourc’h et al., patients of the FMO group were preoxygenated with a 10-cm H_2_O pressure support compared to spontaneous breathing in our study [16]. In addition, the inconsistent use of neuromuscular blocking agents in the preceding study might also confound the efficacy of HFNO [16]. Rodriguez and colleagues recently reported that HFNO did not outperform FMO in preventing severe hypoxemia (SpO_2_ < 80%) during intubation for critically ill obese patients [17]. That study evaluated patients with acute respiratory failure, who were obviously distinctive from anesthetized patients for elective surgery in their respiratory reserve and clinical settings [17]. The present study showed that HFNO as a preoxygenation technique had a greater increase in PaO_2_ in comparison with standard FMO, agreeing with one study [14], but not another [18]. Moreover, the support of HFNO after tracheal extubation has been shown to better prevent postoperative hypoxemia in obese patients compared to oxygen supplement via a Venturi mask [24]. Similarly, a clinical trial recently showed that high-flow nasal cannulas prolong safe apnea time (SpO_2_ > 95%) and reduce the decline in PaO_2_ during apnea among paralyzed obese patients compared to simple nasal prongs [20]. Conversely, Riccio and colleagues claimed that high-flow nasal cannulas did not reduce arterial desaturation during intravenous sedation for obese patients compared to simple nasal cannulas [12]. The conflicting findings in the current literature warrant more effort to investigate the potential clinical benefits and optimal strategy of HFNO in preventing hypoxemia for obese patients undergoing general anesthesia and sedation.

Obese patients have a higher risk of difficult mask ventilation and difficult intubation in comparison with the non-obese population [6,9,25]. The condition means that patients require a rapid sequence intubation (e.g., inability to protect the airway against aspiration or maintain airway patency), making it more complicated to intubate the trachea while preventing oxygen desaturation. Current practice guidelines recommend the use of nasal cannulas as an alternative method to deliver oxygen continuously during the induction of anesthesia in cases of an anticipated difficult airway [26,27]. This is primarily based on studies delivering nasal insufflation of oxygen at a flow of merely 5 L·min^−1^ [28,29]. For rapid sequence intubation, the guidelines recommend the use of gentle mask ventilation before tracheal intubation to prolong nonhypoxic apnea time in patients with poor respiratory reserve or high metabolic requirements [26]. However, the efficacy of high-flow nasal cannulas as a technique of preoxygenation and apneic oxygenation during tracheal intubation has not been fully evaluated by previous works. Studies showed that HFNO improved arterial oxygen saturation and prevented desaturation during awake fiberoptic tracheal intubation compared to FMO in patients with known difficult airways [30]. Two clinical trials reported that HFNO reduced the rate of arterial desaturation below 93% and produced an equivalent blood gas profile to FMO during rapid sequence induction for emergency surgery [31,32]. Taking our study together with previous works, HFNO may be considered as a practicable technique for preoxygenating anesthetized patients during rapid sequence induction [30,31,32].

Some physiological mechanisms have been proposed for the benefits of HFNO in oxygenation and gaseous exchange. First, high-flow nasal cannulas produce a low level of positive airway pressure: 2.7 cm H_2_O at 35 L·min^−1^ of gas flow in healthy volunteers [33]. The apneic oxygenation of HFNO may prolong nonhypoxic apnea time and decrease the occurrence of oxygen desaturation. Second, experimental studies have shown that transnasal gas flow creates supraglottic flow vortices, which interact with cardiogenic oscillations and enhance carbon dioxide clearance in apneic patients [34,35]. The deadspace flushing of HFNO may reduce the rate of carbon dioxide accumulation in anesthetized patients, although our study did not demonstrate such a result.

Attention must be given to some of the limitations of this study. First, the number of patients in this trial was only modest, and some subgroup analyses may have insufficient statistical power. Second, concerns for potential severe hypoxia prevented us from evaluating nonhypoxic apnea time, which is probably a more practical indicator of oxygenation efficacy during tracheal intubation. Third, we did not investigate FMO with positive airway pressure support due to concerns for potential patient discomfort [36]. Fourth, we did not analyze the arterial blood–gas tension immediately after tracheal intubation, because it was difficult to sample the arterial blood whilst performing a recruitment maneuver to promptly manage potential desaturation. Fifth, patients and anesthesiologists could not be blinded, which might have biased the outcome measurement. Sixth, the FMO used in this study could not provide apneic oxygenation during tracheal intubation. Therefore, it remains unclear whether HFNO without apneic oxygenation outperforms FMO or not in preventing desaturation for obese patients. Finally, this trial did not include critically ill patients, such as those with poor respiratory reserve or acute respiratory failure. Consequently, the results cannot be generalized to these patients [17].

## 5. Conclusions

HFNO as a preoxygenation technique reduced the risk of peri-intubation arterial desaturation and achieved a higher PaO_2_ compared to standard FMO in obese patients undergoing general anesthesia. Furthermore, the lowest SpO_2_ during tracheal intubation was higher in patients receiving HFNO compared to FMO. Patient comfort level was similar between the two techniques. These findings indicate that HFNO may be regarded as an alternative technique of preoxygenation for anesthetized obese patients. Our evidence supports the use of HFNO to delay the occurrence of desaturation and to allow more time for tracheal intubation in obese patients, especially in rapid sequence induction or among patients with poor respiratory reserve. Further studies are needed to separately evaluate the efficacy of HFNO during spontaneous breathing or apnea. This will clarify the role of HFNO in preventing peri-intubation desaturation for patients with or without neuromuscular blockades.

## Figures and Tables

**Table 1:** Baseline patient characteristics.

	HFNO *n* = 40		FMO *n* = 40		*p*
Age, years	36.7	9.2	36.7	9.1	0.9806
Sex, male	20	50.0	18	45.0	0.6543
Body mass index, linear, kg·m^−2^	40.3	36.6–43.8 (30.9–51.6)	40.0	35.1–47.1 (31.9–59.2)	0.5572
Body mass index, binary, kg·m^−2^					0.8230
<40	19	47.5	20	50.0	
≥40	21	52.5	20	50.0	
Waist circumference, cm	124.0	13.0	126.5	15.4	0.4468
ASA physical status					0.8230
Class II	19	47.5	20	50.0	
Class III	21	52.5	20	50.0	
Modified Mallampati score					0.4144
Class I	11	27.5	7	17.5	
Class II	7	17.5	13	32.5	
Class III	13	32.5	11	27.5	
Class IV	9	22.5	9	22.5	
Current cigarette smoking	20	50.0	14	35.0	0.1748
Current alcohol drinking	7	17.5	7	17.5	>0.9999
Coexisting disease					
Hypertension	14	35.0	13	32.5	0.8131
Diabetes mellitus	8	20.0	6	15.0	0.5562
Chronic kidney disease	2	5.0	0	0	0.4937
Fatty liver	34	85.0	30	75.0	0.2636
Obstructive sleep apnea	13	32.5	19	47.5	0.1709
Preoperative blood test					
Hemoglobin, g·dL^−1^	14.4	13.7–15.6 (10.8–17.8)	14.7	13.8–15.4 (8.7–17.4)	0.9654
Creatinine, mg·dL^−1^	0.74	0.61–0.88 (0.40–1.51)	0.74	0.61–0.87 (0.47–1.11)	0.9424
eGFR, mL·min·1.73 m^−2^	110.7	92.6–129.6 (53.9–189.9)	115.1	92.3–125.4 (74.5–166.5)	0.8211
Sodium, mmol·L^−1^	139	137–140 (134–144)	138	137–140 (130–145)	0.7187
Potassium, mmol·L^−1^	3.9	3.8–4.1 (3.3–4.4)	3.9	3.6–4.1 (3.4–4.4)	0.8130
Alanine aminotransferase, U·L^−1^	30	24–54 (15–242)	32	25–41 (12–159)	0.9501
Aspartate aminotransferase, U·L^−1^	36	24–47 (12–91)	31	22–48 (16–305)	0.5410

Values are mean ± standard deviation, counts (percent), or median (interquartile range; minimum and maximum). Abbreviations: ASA, American Society of Anesthesiologists; eGFR, estimated glomerular filtration rate; FMO, facemask oxygenation; HFNO, high-flow nasal oxygenation.

**Table 2:** Baseline oxygen saturation and arterial blood gas data, intravenous anesthetic agents, and intubation parameters.

	HFNO *n* = 40		FMO *n* = 40		*p*
SpO_2_ in room air, %	97	96–98 (95–100)	97	96–98 (93–99)	0.8244
PaO_2_ in room air, mm Hg	90	81–97 (65–133)	84	76–93 (61–181)	0.1104
PaCO_2_ in room air, mm Hg	39.1	37.3–42.1 (30.1–49.6)	41.6	38.1–43.9 (33.4–55.2)	0.0604
Intravenous anesthetics					
Lidocaine, mg	80	80–100 (60–100)	80	80–100 (60–100)	0.6614
Dexamethasone, mg	5	5–5 (5–5)	5	5–5 (5–5)	>0.9999
Glycopyrrolate, mg	0.2	0.2–0.2 (0.2–0.2)	0.2	0.2–0.2 (0.2–0.2)	>0.9999
Fentanyl, μg	200	150–200 (150–250)	200	150–200 (100–250)	0.2038
Propofol, mg	200	150–200 (120–200)	200	150–200 (130–200)	0.7685
Rocuronium, mg	98	83–125 (60–200)	98	90–100 (60–160)	0.8428
First-attempt success of intubation	40	100.0	38	95.0	0.4937
Time to successful intubation, s	23	18–31 (11–62)	24	19–34 (12–130)	0.5470
Need to use a video laryngoscope	0	0	0	0	NA

Values are median (interquartile range; minimum and maximum). Abbreviations: FMO, facemask oxygenation; HFNO, high-flow nasal oxygenation; NA, not applicable; PaO_2_, partial pressure of arterial oxygen; PaCO_2_, partial pressure of arterial carbon dioxide; SpO_2_, peripheral capillary oxygen saturation.

**Table 3:** Study outcomes.

	HFNO *n* = 40		FMO *n* = 40		*p*
Desaturation (lowest SpO_2_ < 92%)	2	(5.0)	10	(25.0)	0.0122
Lowest SpO_2_ during intubation, %	99	97–100 (81–100)	96	92–100 (80–100)	0.0150
PaO_2_ after preoxygenation, mm Hg	476	390–541 (260–620)	397	351–456 (210–632)	0.0010
PaCO_2_ after preoxygenation, mm Hg	41.5	36.0–43.9 (22.7–53.1)	42.2	35.9–47.3 (26.3–58.1)	0.2076
SpO_2_ after preoxygenation, %	100	100–100 (98–100)	100	100–100 (97–100)	0.9933
Comfort level on 10-point Likert scale	8	7–10 (2–10)	8	8–9 (6–10)	0.7118

Values are counts (percent) or median (interquartile range; minimum and maximum). Abbreviations: FMO, facemask oxygenation; HFNO, high-flow nasal oxygenation; PaO_2_, partial pressure of arterial oxygen; PaCO_2_, partial pressure of arterial carbon dioxide; SpO_2_, peripheral capillary oxygen saturation.

**Table 4:** Sensitivity analyses for peri-intubation oxygen desaturation.

	HFNO *n* = 40		FMO *n* = 40		*p*
Desaturation (lowest SpO_2_ < 92%)	2	5.0	10	25.0	0.0122
Desaturation (lowest SpO_2_ < 95%)	7	17.5	15	37.5	0.0452
Desaturation (lowest SpO_2_ < 90%)	1	2.5	7	17.5	0.0568
Desaturation (lowest SpO_2_ < 92%), excluding patients requiring more than one intubation attempt	2	5.0	8	21.1	0.0448

Values are counts (percent). Abbreviations: FMO, facemask oxygenation; HFNO, high-flow nasal oxygenation; SpO_2_, peripheral capillary oxygen saturation.

**Table 5:** Subgroup analyses by body mass index and obstructive sleep apnea.

	HFNO *n* = 40		FMO *n* = 40		*p*
Desaturation (lowest SpO_2_ < 92%)					
All	2	5.0	10	25.0	0.0122
Body mass index ≥40 kg·m^−2^	1	4.8	7	35.0	0.0205
Body mass index <40 kg·m^−2^	1	5.3	3	15.0	0.6050
Obstructive sleep apnea	1	7.7	4	21.1	0.6247
No obstructive sleep apnea	1	3.7	6	28.6	0.0335
Lowest SpO_2_ during intubation, %					
All	99	97–100 (81–100)	96	92–100 (80–100)	0.0150
Body mass index ≥40 kg·m^−2^	99	96–100 (81–100)	95	89–99 (80–100)	0.0475
Body mass index <40 kg·m^−2^	98	98–100 (90–100)	96	95–100 (85–100)	0.1222
Obstructive sleep apnea	98	96–99 (81–100)	97	92–100 (80–100)	0.5106
No obstructive sleep apnea	99	97–100 (90–100)	95	91–99 (81–100)	0.0146
PaO_2_ after preoxygenation, mmHg					
All	476	390–541 (260–620)	397	351–456 (210–632)	0.0010
Body mass index ≥40 kg·m^−2^	468	395–546 (260–605)	394	329–480 (210–632)	0.0696
Body mass index <40 kg·m^−2^	505	385–537 (278–620)	399	360–422 (237–495)	0.0090
Obstructive sleep apnea	470	395–536 (341–559)	402	357–433 (210–632)	0.0445
No obstructive sleep apnea	482	385–544 (260–620)	383	351–467 (237–540)	0.0091

Values are counts (percent) or median (interquartile range; minimum and maximum). Abbreviations: FMO, facemask oxygenation; HFNO, high-flow nasal oxygenation; PaO_2_, partial pressure of arterial oxygen; SpO_2_, peripheral capillary oxygen saturation.

## Data Availability

The data presented in this study are available on request from the corresponding author. The data are not publicly available due to the regulations of the Institutional Review Board.
